# Atrial Cardiomyopathy in Atrial Fibrillation: A Multimodal Diagnostic Framework

**DOI:** 10.3390/diagnostics15101207

**Published:** 2025-05-10

**Authors:** Paschalis Karakasis, Panayotis K. Vlachakis, Panagiotis Theofilis, Nikolaos Ktenopoulos, Dimitrios Patoulias, Barbara Fyntanidou, Antonios P. Antoniadis, Nikolaos Fragakis

**Affiliations:** 1 Second Department of Cardiology, Hippokration General Hospital, Aristotle University of Thessaloniki, 54642 Thessaloniki, Greece; aantoniadis@gmail.com; 2 First Cardiology Department, School of Medicine, Hippokration General Hospital, National and Kapodistrian University of Athens, 11527 Athens, Greece; vlachakispanag@gmail.com (P.K.V.); panos.theofilis@hotmail.com (P.T.); nikosktenop@gmail.com (N.K.); 3 Second Propedeutic Department of Internal Medicine, Faculty of Medicine, School of Health Sciences Aristotle, University of Thessaloniki, 54642 Thessaloniki, Greece; dipatoulias@gmail.com; 4 Emergency Department, AHEPA University General Hospital, Aristotle University of Thessaloniki, 54642 Thessaloniki, Greece; bfyntan@yahoo.com

**Keywords:** atrial cardiomyopathy, atrial fibrillation, cardiac imaging, electrocardiography, electroanatomic mapping, biomarkers, atrial substrate

## Abstract

Atrial fibrillation (AF) is increasingly recognized as the clinical manifestation of an underlying atrial disease process rather than a purely electrical disorder. This evolving paradigm has given rise to the concept of atrial cardiomyopathy (AtCM), encompassing structural, electrical, contractile, and molecular remodeling of the atrial myocardium that contributes to AF initiation, maintenance, and progression. Although consensus definitions of AtCM now exist, its integration into clinical practice remains limited, with AF management still largely guided by arrhythmic patterns rather than substrate characterization. This review synthesizes current diagnostic strategies for AtCM within the context of AF, emphasizing a multimodal approach. We outline advances in cardiac imaging—including echocardiography, cardiac magnetic resonance, and computed tomography—for detailed assessment of atrial morphology, function, and fibrosis. Electroanatomic mapping is discussed as a key invasive tool for substrate localization, while electrocardiographic indices such as P-wave morphology and dispersion serve as accessible surrogates of electrical remodeling. In parallel, we examine the role of circulating biomarkers and emerging genomic, transcriptomic, and epigenomic markers in refining disease phenotyping. Despite promising progress, significant challenges remain. Standardization of imaging protocols, validation of biomarker thresholds, and integration of artificial intelligence tools are needed to enhance clinical utility. A diagnostic framework informed by atrial substrate assessment may support more tailored therapeutic decision-making in AF. Future research should prioritize the harmonization of diagnostic criteria and explore how substrate profiling in AF may refine risk stratification and improve clinical outcomes.

## 1. Introduction

Atrial fibrillation (AF) is the most prevalent sustained arrhythmia encountered in clinical practice and is associated with increased morbidity, mortality, and healthcare burden [1,2,3,4,5,6,7]. AF has long been conceptualized as an isolated electrical disturbance; however, growing evidence underscores the primacy of atrial structural and functional remodeling in its pathogenesis and progression [8]. This reconceptualization has culminated in the emergence of atrial cardiomyopathy (AtCM)—a term denoting a constellation of architectural, contractile, and electrophysiologic alterations of the atrial myocardium that may exist independently of, or in conjunction with, AF [8,9]. Recognizing AtCM as the arrhythmogenic substrate underlying AF reframes the disorder not merely as an arrhythmia but as the clinical expression of a more diffuse and heterogeneous atrial disease process [10].

Consensus definitions now describe AtCM as any complex of atrial abnormalities capable of producing clinically relevant manifestations, including atrial arrhythmias, thromboembolism, or atrial failure [8]. Recent expert consensus from the European Association of Cardiovascular Imaging (EACVI) and the European Heart Rhythm Association (EHRA) has further emphasized the central role of left atrial structure and function in this context, advocating for comprehensive multimodality imaging to guide clinical assessment and management strategies [11]. Importantly, AF may result from, exacerbate, or even mask the underlying substrate of AtCM, contributing to a bidirectional and progressive disease process. Histopathologic studies, translational investigations, and clinical imaging have each identified diverse mechanisms—fibrosis, inflammation, oxidative stress, adipose infiltration, and aging—that converge to produce electrophysiological instability and mechanical dysfunction in the atria [12,13,14,15].

A staging framework for AtCM was recently proposed in a clinical consensus statement by the EHRA, together with HRS, APHRS, and LAHRS, integrating structural, functional, and electrophysiological criteria [8]:

1. Early (subclinical) stage: characterized by the presence of subtle atrial abnormalities identifiable through advanced imaging or electrophysiologic assessment, in the absence of overt arrhythmia or mechanical dysfunction. These changes are often asymptomatic and represent early atrial remodeling;
2. Intermediate (clinically overt) stage: defined by measurable structural alterations (e.g., atrial enlargement), impaired atrial contractility, or elevated circulating biomarkers such as B-type natriuretic peptide (BNP) or atrial natriuretic peptide (ANP). Atrial fibrillation may be present at this stage, indicating clinically significant substrate dysfunction;
3. Advanced (severe) stage: reflects substantial deterioration of atrial function, including marked reduction in left atrial ejection fraction (≤35%), low atrial appendage flow velocities (≤20 cm/s), or severely impaired strain parameters. This stage may also involve extensive structural remodeling such as dense interstitial fibrosis (≥35% of atrial wall volume), fatty or amyloid infiltration, or active inflammation. Severe left atrial enlargement (diameter ≥ 5.0 cm or volume index ≥ 50 mL/m^2^) and the presence of long-standing persistent or permanent atrial fibrillation are common.

**Figure 1:**
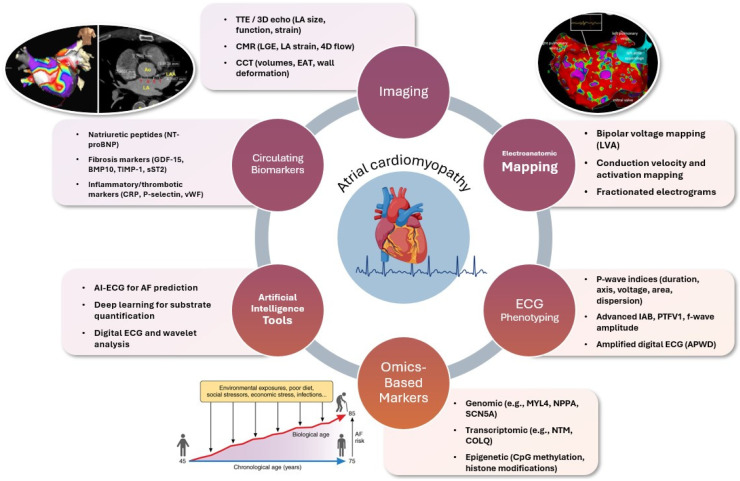
Integrated multimodal diagnostic framework for atrial cardiomyopathy in atrial fibrillation. This schematic illustrates a comprehensive approach to diagnosing atrial cardiomyopathy (AtCM) in the context of atrial fibrillation (AF), emphasizing the convergence of structural, electrical, functional, and molecular assessments. Central to the diagram is AtCM, surrounded by six key diagnostic domains: (1) imaging modalities (echocardiography, cardiac magnetic resonance, and computed tomography) provide detailed assessment of atrial size, function, fibrosis, and wall deformation; (2) electroanatomic mapping offers invasive evaluation of low-voltage areas, conduction velocity, and electrogram complexity; (3) ECG phenotyping captures electrical remodeling through P-wave indices, interatrial block, and f-wave morphology; (4) circulating biomarkers reflect hemodynamic stress, inflammation, and fibrosis; (5) omics-based markers—including genomic, transcriptomic, and epigenetic alterations—offer molecular insight into atrial remodeling; and (6) artificial intelligence tools enhance diagnostic precision through advanced ECG interpretation and substrate quantification. Together, these modalities contribute to a substrate-guided framework for risk stratification, therapy selection, and progression monitoring in AF. Abbreviations: TTE—transthoracic echocardiography; 3D—three-dimensional; CMR—cardiac magnetic resonance; LGE—late gadolinium enhancement; LA—left atrium; CCT—cardiac computed tomography; EAT—epicardial adipose tissue; LVA—low-voltage area; ECG—electrocardiogram; IAB—interatrial block; PTFV1—P-wave terminal force in lead V1; APWD—amplified P-wave duration; AI—artificial intelligence; AF—atrial fibrillation; NT-proBNP—N-terminal pro–B-type natriuretic peptide; GDF-15—growth differentiation factor-15; BMP10—bone morphogenetic protein 10; TIMP-1—tissue inhibitor of metalloproteinase-1; sST2—soluble suppression of tumorigenicity-2; CRP—C-reactive protein; vWF—von Willebrand factor; MYL4—myosin light chain 4; NPPA—natriuretic peptide A; SCN5A—sodium voltage-gated channel alpha subunit 5; NTM—neurotrimin; COLQ—collagen-like tail subunit of asymmetric acetylcholinesterase; CpG—cytosine–phosphate–guanine.

Despite this conceptual shift, the clinical integration of AtCM remains underdeveloped and has yet to be fully translated into routine practice [16,17]. Current AF management strategies still largely rely on rhythm classification, without explicit characterization of atrial pathology. Yet, advances in cardiac imaging, electrocardiography, electroanatomic mapping, and circulating biomarkers now offer an unprecedented opportunity to non-invasively assess atrial structure and function (Figure 1). The synthesis of these diagnostic modalities may enable early identification of AtCM, more precise risk stratification for AF-related complications, and refined targeting of therapeutic strategies—including ablation, anticoagulation, and upstream disease modification.

In this review, we summarize current approaches to diagnosing AtCM in the setting of AF, drawing on advances in imaging, electroanatomic mapping, electrocardiography, biomarkers, and omics.

## 2. Advancements in Diagnostic Imaging

A comprehensive, multimodality assessment of atrial morphology and function through advanced cardiac imaging techniques is essential for the accurate diagnosis of atrial cardiomyopathy and for delineating the extent of atrial structural and functional impairment [18].

### 2.1. Advanced Atrial Imaging in the Diagnostic Evaluation of AtCM

AtCM can be comprehensively assessed using a range of imaging modalities, including transthoracic echocardiography (TTE), cardiac magnetic resonance (CMR), and cardiac computed tomography (CCT). Both echocardiography and CMR facilitate dynamic quantification of atrial volumes throughout the cardiac cycle, whereas CCT provides precise volumetric measurements at a fixed point in time [19]. Among echocardiographic indices, the left atrial volume index (LAVI) is a particularly well-established marker of atrial remodeling. Easily obtainable via TTE, LAVI is reproducible and widely adopted in clinical practice [20]. A LAVI ≤ 34 mL/m^2^ is considered normal for both sexes, while values of 35–41 mL/m^2^, 42–48 mL/m^2^, and >48 mL/m^2^ correspond to mild, moderate, and severe atrial enlargement, respectively [20]. It is strongly associated with the presence, progression, and recurrence of AF, and remains central to echocardiographic risk stratification [21]. Beyond static volumetric measures, recent studies have introduced the left atrioventricular coupling index (LACI) as a dynamic marker of atrial–ventricular interaction [22,23]. Defined as the ratio of LA to LV end-diastolic volume, LACI reflects atrioventricular mechanical coupling and has demonstrated strong prognostic value. In the Multi-Ethnic Study of Atherosclerosis (MESA), both an elevated LACI (>30%) and its annualized increase (>1.5% per year) were independently associated with incident AF, offering superior predictive discrimination compared to conventional structural indices [23]. These findings suggest that LACI may provide complementary insights into atrial remodeling and functional integration within the left heart.

Three-dimensional TTE mitigates the limitations of atrial foreshortening inherent to two-dimensional approaches, thereby enhancing anatomical fidelity [19]. The left and right atrial appendages are more accurately delineated using transesophageal echocardiography (TEE) and CCT, which offer superior spatial resolution for these anatomically complex structures. In addition, CCT has been validated for the assessment of epicardial adipose tissue (EAT) and is now increasingly augmented by deep learning algorithms to improve image interpretation and prognostic insights [24]. Notably, alterations in the morphology of the left atrial (LA) roof, as visualized on CT, have been associated with the emergence of non–pulmonary vein (non-PV) arrhythmogenic substrates in patients undergoing atrial fibrillation (AF) ablation [25,26]. Furthermore, pre-procedural assessment of regional LA wall deformation via CCT has demonstrated superior predictive accuracy for low-voltage areas compared to traditional remodeling indices, underscoring its potential value in procedural planning and risk stratification [27]. CMR has been instrumental in delineating atrial fibrotic remodeling through late gadolinium enhancement imaging, while also facilitating the assessment of intra-atrial flow dynamics via 4D flow-derived velocity mapping [28,29].

Moreover, although not widely implemented in routine clinical practice, molecular positron emission tomography (PET) imaging represents a novel approach for characterizing atrial inflammation and fibrosis. Increased atrial uptake of 18F-fluorodeoxyglucose (18F-FDG) has been observed in patients with AF and in those with cardiac sarcoidosis, where it may predict subsequent arrhythmia onset [30,31]. More recently, 68Ga–fibroblast activation protein inhibitor (68Ga-FAPI) PET imaging has been employed to detect elevated fibroblast activation in the atria of individuals with AF or following pulmonary vein isolation, suggesting its potential utility in substrate characterization [32,33]. Although atrial activity is infrequently reported in oncologic PET/CT imaging, incidental atrial uptake has been associated with a heightened risk of AF and thromboembolic complications, underscoring its possible relevance in cardio-oncology [30]. These findings have been substantiated by a recent prospective case-control study demonstrating that right atrial 18F-FDG uptake was significantly greater in patients with persistent AF and independently associated with inflammatory burden [34]. Complementary evidence from a systematic review and meta-analysis further confirmed a strong association between atrial FDG uptake and AF, with markedly elevated odds of both left and right atrial involvement in affected patients [35]. Moreover, hybrid PET-MRI imaging using FAPI tracers has enabled visualization of atrial fibroblast activation that correlates with histological fibrosis, particularly in persistent AF [36]. Preclinical work utilizing 11C-hydroxyephedrine PET has also revealed sympathetic denervation accompanying fibrosis, emphasizing the multifaceted remodeling processes underpinning AF [37]. While these advances position PET imaging as a powerful research tool for substrate phenotyping, its clinical utility remains investigational and awaits further validation. A comparative overview of the technical capabilities and diagnostic applications of each modality in the context of atrial evaluation is presented in Table 1.

### 2.2. Quantitative Assessment of Atrial Chamber Dimensions and Functional Dynamics

The atria perform a series of dynamic and physiologically distinct roles throughout the cardiac cycle, functioning sequentially as a reservoir for pulmonary or systemic venous return during ventricular systole, as a passive conduit during early diastole, and as an active booster pump during atrial systole [38,39]. These three functional phases are quantitatively represented by specific atrial volumetric parameters: (i) the maximum atrial volume, measured at end-systole and corresponding to the reservoir phase; (ii) the pre-atrial contraction volume, assessed in mid-diastole during the conduit phase; and (iii) the minimum atrial volume, determined in late diastole following atrial contraction, indicative of booster–pump function [38,39].

Derived indices of atrial function integrate these volume measurements to characterize global atrial performance [40,41]. The total atrial emptying fraction and expansion index are calculated based on the difference between maximal and minimal atrial volumes, reflecting overall contractile reserve and compliance [41]. The passive emptying fraction is derived from the difference between maximal and pre-atrial contraction volumes, corresponding to conduit function, while the active emptying fraction quantifies booster pump activity by comparing pre-atrial contraction and minimal volumes [42].

Beyond volumetric assessment, atrial strain analysis, predominantly performed using TTE and CMR, provides a sensitive measure of myocardial deformation during the reservoir, conduit, and contractile phases [43]. In this context, reservoir strain, conduit strain, and contractile strain offer mechanistic insights into atrial compliance, passive filling, and active contraction, respectively [43].

CCT has recently emerged as a promising modality for the comprehensive evaluation of atrial structure and function in AtCM. Owing to its superior spatial resolution relative to other imaging techniques, CCT enables detailed anatomical and functional characterization, facilitating advanced phenotyping of atrial remodeling [44]. Normative data for left and right atrial size and function across the cardiac cycle have been established using TTE, CMR, and CCT [20,45,46,47,48,49,50,51]. Notably, recent evidence suggests that a left atrial reservoir strain below 18% may serve as a surrogate marker of elevated left ventricular filling pressures in the context of heart failure. However, definitive thresholds to demarcate irreversible atrial dysfunction in AtCM have not yet been established (Table 2).

### 2.3. Late Gadolinium-Enhanced Cardiac Magnetic Resonance for Left Atrial Tissue Characterization

Late gadolinium-enhanced cardiac magnetic resonance (LGE-CMR) has become a cornerstone in the diagnostic and prognostic evaluation of ventricular cardiomyopathies, primarily by identifying myocardial regions with expanded interstitial space or delayed gadolinium washout [52]. Traditionally, LGE serves as a surrogate for myocardial fibrosis in both chronic ischemic injury and a spectrum of non-ischemic cardiomyopathies [52]. However, it may also reflect other pathological processes such as interstitial edema, inflammatory infiltration—as seen in acute myocarditis or cardiac sarcoidosis—or amyloid protein deposition, all of which alter the tissue composition and gadolinium kinetics [52,53].

In recent years, efforts have been made to adapt LGE-CMR techniques for the evaluation of the left atrium, particularly to visualize and quantify atrial fibrotic remodeling [27]. Despite these advances, the application of LGE-CMR to atrial tissue remains fraught with methodological challenges [54,55]. These include the absence of universally accepted imaging protocols, heterogeneity in post-processing algorithms, and inherent limitations related to the thin atrial wall, which compromises spatial resolution [54,55]. Furthermore, access to high-quality LA LGE imaging remains largely restricted to specialized research centers.

Comparative analyses of existing LGE-CMR methodologies have demonstrated marked variability in the reported burden and spatial distribution of fibrosis, both globally and regionally [56,57]. Importantly, conventional LGE imaging is optimized to highlight focal areas of fibrosis by nulling normal myocardium [58]. Consequently, it lacks the sensitivity to detect diffuse interstitial fibrosis—a significant drawback, particularly given autopsy evidence suggesting that atrial fibrosis in AF is often a widespread, rather than localized, process [59,60,61]. Thus, LGE-positive regions in the atria may correspond to advanced fibrotic lesions or potentially represent nonfibrotic pathological changes.

Despite these limitations, LGE-CMR has yielded important mechanistic insights. Studies have shown that electrical rotors—key drivers of AF—tend to anchor at fibrotic–nonfibrotic interfaces, underscoring the arrhythmogenic potential of structurally heterogeneous tissue [62]. Moreover, the extent of LA fibrosis identified by LGE has been linked to higher rates of AF recurrence following catheter ablation [63] and an elevated risk of stroke [64]. Nonetheless, recent randomized controlled trials investigating LGE-guided ablation strategies have failed to demonstrate superior outcomes in arrhythmia suppression when targeting fibrotic regions in addition to pulmonary vein isolation, suggesting that the clinical utility of LGE-CMR–guided substrate modification remains uncertain and may require further refinement [65,66].

### 2.4. Electroanatomic Mapping

Electroanatomic mapping has become an essential tool for substrate characterization in AtCM, particularly during catheter ablation procedures. This technique enables the acquisition of detailed voltage maps, activation sequence patterns, conduction velocity profiles, and the identification of fractionated electrograms, thereby facilitating a comprehensive electrophysiological assessment of the atrial substrate [57,67,68,69].

One of the principal applications of electroanatomic mapping in AtCM involves the delineation of low-voltage areas (LVAs), typically defined as regions exhibiting bipolar endocardial voltage amplitudes below 0.5 mV [68]. These regions are commonly interpreted as surrogates for underlying atrial fibrosis. Studies have demonstrated that the extent and distribution of LVAs are correlated with atrial structural remodeling, including increased left atrial volume and the presence of cardiovascular comorbidities. Importantly, the voltage attenuation observed in these maps often represents a diffuse electrophysiological phenomenon, with LVAs reflecting localized manifestations within a broader context of global voltage reduction [68]. Currently, no consensus exists regarding definitive cutoff values for atrial fibrosis, and the extent of LVA exhibits only weak correlation with clinical AF history [70,71,72,73,74]. Although combined use of electroanatomic mapping and advanced imaging may enhance substrate identification in atrial arrhythmias [75], substantial variability persists. The development of non-invasive atrial mapping techniques to detect and quantify AtCM remains an area of active and promising investigation [76].

Histopathological investigations have supported the link between reduced atrial voltage and fibrotic remodeling; however, recent biopsy-based evidence indicates that voltage diminution is not exclusively attributable to fibrosis [69]. Other structural alterations—including expanded extracellular matrix volume, loss of myofibrils, diminished cardiomyocyte nuclear density, and amyloid infiltration—have also been implicated in voltage reduction [69]. These findings highlight the multifactorial and heterogeneous nature of atrial substrate pathology in AtCM and suggest that LVA mapping captures a complex spectrum of tissue alterations beyond fibrosis alone.

In addition to catheter-based electroanatomic mapping, emerging non-invasive current-based techniques such as magnetocardiography (MCG) offer novel avenues for atrial substrate assessment [77]. MCG reconstructs pseudo-current distributions derived from cardiac magnetic field measurements and provides contact-free characterization of atrial electrophysiology [77]. In a study by Her et al. [78], MCG was able to detect impaired left atrial (LA) pseudo-current responses during exercise in patients with paroxysmal atrial fibrillation (PAF), compared to healthy controls and endurance athletes [78]. Notably, the exercise-induced change in the LA pseudo-current demonstrated high diagnostic accuracy for PAF, with a sensitivity of 77%, specificity of 92%, and an area under the receiver-operating curve of 0.896. These findings suggest that MCG may serve as a valuable adjunctive tool for identifying functional atrial abnormalities in patients with early-stage AF or at risk for atrial cardiomyopathy.

### 2.5. Imaging Predictors of AF Onset

To date, no AF management strategy is guided exclusively by cardiac imaging findings. However, accumulating evidence from large-scale cohort studies employing TTE and CMR has demonstrated that structural and functional remodeling of the LA—manifested as increased LA volumes and reduced LA functional indices—is significantly associated with the future development of AF [79,80,81,82,83]. In addition to LA parameters, right atrial (RA) enlargement has emerged as an independent predictor of incident AF. Data from a diverse, multi-ethnic population cohort revealed that elevated RA volume indices remained significantly associated with new-onset AF even after adjusting for cardiovascular comorbidities and concomitant LA characteristics [84].

CCT-based quantification of epicardial adipose tissue (EAT) has further expanded the imaging toolkit for AF risk prediction. Increased EAT mass, as measured by CCT, has been independently linked to a heightened risk of incident AF [85,86]. Moreover, EAT volume demonstrates a strong association with both paroxysmal and persistent AF phenotypes, independent of conventional risk factors [87]. Functional impairment and dilation of the LA have also been correlated with subclinical atrial ectopy. Specifically, both reduced LA function and LA enlargement are associated with an increased frequency of premature atrial contractions on prolonged ambulatory electrocardiographic monitoring—an established harbinger of AF [88,89].

### 2.6. Recurrent AF

Morphological remodeling of the left atrial (LA) roof, as characterized by CT, has been implicated in the emergence of non–pulmonary vein (non-PV) arrhythmogenic substrates among patients undergoing AF ablation [25,26]. Additionally, pre-procedural assessment of regional LA wall deformation using cardiac CT has demonstrated superior predictive accuracy for the presence of low-voltage areas (LVAs) compared to conventional structural remodeling markers, underscoring its potential utility in procedural planning [27].

LGE-CMR–derived quantification of LA fibrosis has also been shown to independently predict arrhythmia recurrence following AF ablation, reinforcing its relevance in substrate characterization [63]. Nevertheless, two recent randomized controlled trials evaluating fibrosis-guided ablation strategies failed to demonstrate a significant reduction in AF recurrence when LA fibrosis-targeted ablation was performed in addition to standard pulmonary vein isolation (PVI), compared with PVI alone [65,66].

These findings may be attributable to several key factors. One trial postulated that the absence of clinical benefit reflected a relatively low overall fibrotic burden within the study cohort, limiting the efficacy of adjunctive substrate modification [65]. The other highlighted ongoing technical limitations, particularly the lack of standardized imaging protocols, reproducible fibrosis quantification methods, and validated ablation endpoints specific to fibrotic tissue [66]. Furthermore, the authors hypothesized that atrial fibrosis is not a homogeneous entity; rather, distinct histopathological subtypes—such as interstitial versus reparative fibrosis—may exhibit differential contributions to the arrhythmogenic substrate, complicating efforts to tailor ablation strategies solely on the basis of LGE distribution [66].

### 2.7. Atrial Remodeling and Reverse Remodeling

Cardiac imaging offers valuable prognostic insights into the potential for reverse atrial remodeling following therapeutic interventions for AF [90]. It enables detailed evaluation of both structural and functional recovery, including improvements in atrioventricular valve competence—particularly in cases of functional mitral and tricuspid regurgitation—after restoration of sinus rhythm [91]. In a mechanistic study using CCT, Huang et al. [92] reported that atrial remodeling may commence rapidly, with measurable increases in left and right atrial volumes occurring after just 6 min of atrial high-rate episodes (AHREs). When the duration of AHRE exceeded 6 h, biatrial contractile dysfunction became evident. These findings suggest a time-dependent progression of atrial remodeling, where structural dilation precedes functional decline, and underscore the potential of imaging to detect early changes and guide therapeutic timing.

### 2.8. Imaging and Risk Stratification

CCT-based evaluations of atrial remodeling in the context of atrial fibrillation (AF)-related stroke have demonstrated that advanced age and diminished atrial booster–pump function are independently associated with stroke occurrence [93]. These findings support the potential utility of substrate-oriented imaging parameters in refining thromboembolic risk stratification among patients with AF [93]. In parallel, the assessment of LA strain has been shown to enhance the predictive accuracy for thromboembolic events in AF and may aid in identifying the presence of LA or left atrial appendage (LAA) thrombus, as detected by transesophageal echocardiography [28,94,95]. Furthermore, variations in LAA morphology and increased LAA volume have been correlated with a prior history of ischemic stroke or transient ischemic attack, reinforcing the link between anatomical features and thromboembolic vulnerability [96,97]. AF itself is associated with globally impaired flow dynamics within both the LA and LAA, contributing to stasis and thrombus formation [98].

Irrespective of AF history, individuals at intermediate-to-high thromboembolic risk demonstrate altered intra-atrial flow dynamics on CMR, indicating the presence of functional atrial abnormalities prior to clinically manifested arrhythmia [98,99]. Furthermore, elevated atrial FDG uptake on PET imaging has been associated with increased stroke prevalence among patients with AF, implicating atrial metabolic or inflammatory activity in the pathophysiology of thromboembolic events [100].

Electroanatomic mapping findings have reinforced these associations, with the presence of LVA being linked to both clinical stroke history and subclinical SCI [101]. Additionally, LVA has shown significant associations with coexisting heart failure with reduced systolic function in individuals with non-valvular AF, suggesting its utility as a marker of more advanced atrial remodeling [102].

In a contemporary observational cohort of 1488 patients undergoing AF ablation, both the presence and burden of LVA were independently predictive of adverse long-term outcomes, including all-cause mortality, heart failure, and stroke, independent of AF recurrence or other confounding clinical variables [103]. These data collectively underscore the potential of cardiac imaging to detect subclinical atrial dysfunction and support its integration into the diagnostic framework of AtCM. Its emerging prognostic value further positions it as a promising tool for refined risk stratification and therapeutic decision-making, warranting continued investigation [100].

## 3. Biomarkers, Omics, and ECG Parameters

A diverse array of biomarkers from blood, urine, imaging, and ECG have been proposed as surrogates for identifying an underlying AtCM phenotype predictive of AF onset and progression. When combined with ECG, electrophysiological, or echocardiographic data, blood-based markers offer a multidimensional strategy for defining AtCM. Spanning electrophysiological, structural, hemodynamic, serological, and genetic domains, these biomarkers have all been linked to thromboembolic risk—even in the absence of manifest AF.

Consequently, such biomarkers have been adopted as diagnostic criteria in studies exploring the relationship between AtCM and cerebrovascular events. For instance, the ARCADIA trial [104] enrolled patients aged ≥45 years with embolic stroke of undetermined source (ESUS) and AtCM, as defined by the presence of at least one of the following: P-wave terminal force > 5000 µV·ms in ECG lead V1, NT-proBNP > 250 pg/mL, or an indexed left atrial diameter ≥ 3 cm/m^2^ on echocardiography. Notably, the trial did not establish the efficacy of biomarker-guided identification of AtCM in selecting patients with cryptogenic stroke who would benefit from oral anticoagulation [105].

The ARIC study further examined echocardiographic parameters of LA function—namely reservoir, conduit, and contractile strain—as well as the LA volume index in relation to incident ischemic stroke [106]. Subsequent analysis from the same cohort revealed that the presence of AtCM, defined by the P-wave terminal force, NT-proBNP, and LA volume index, was also significantly associated with an elevated risk of dementia, independent of AF or prior stroke [107].

### 3.1. Blood Biomarkers

Although biomarkers associated with AF have been extensively investigated [108,109], comparatively limited attention has been given to those specifically indicative of AtCM in the absence of AF [110]. Notably, a substantial number of biomarkers have been evaluated in both contexts, underscoring their limited specificity for distinguishing between AF-driven and substrate-driven pathology. In certain cases, the transition from paroxysmal to persistent AF is interpreted as a clinical marker of progressive atrial remodeling, and several biomarkers have been explored in this setting to elucidate underlying substrate alterations.

Natriuretic peptides, traditionally linked to heart failure pathophysiology [111], have also been independently associated with the risk of incident AF and its clinical sequelae, including thromboembolic events [112,113]. Similarly, cardiac troponin T (cTnT)—a well-established biomarker of myocardial injury—was identified as a predictor of cardioembolic stroke in the ARIC cohort [114].

Beyond markers of myocardial stress and necrosis, biomarkers of extracellular matrix remodeling have also demonstrated relevance in the context of AF [115]. Elevated levels of soluble suppression of tumorigenicity-2 (sST2) and tissue inhibitor of matrix metalloproteinase-1 (TIMP-1) have been associated with AF progression, independent of conventional clinical risk factors and other circulating biomarkers [115]. Fibrosis-related markers have additionally shown prognostic utility in predicting AF recurrence following electrical cardioversion [110].

More recently, bone morphogenetic protein 10 (BMP10)—a protein predominantly expressed in atrial myocytes—has emerged as a potential biomarker of arrhythmic vulnerability. Elevated serum levels of BMP10 have been linked to AF recurrence following catheter ablation [116]. Furthermore, observational cohort studies in AF populations have demonstrated associations between biomarker elevations and increased risks of ischemic stroke [117], all-cause mortality, and major adverse cerebrovascular and cardiovascular events [118].

C-reactive protein (CRP) has been consistently associated with both the presence and burden of AF in nonsurgical populations, implicating systemic inflammation as a key driver of atrial remodeling [119]. Additionally, elevated levels of von Willebrand factor and soluble P-selectin—markers of endothelial dysfunction and platelet activation—have been linked to increased stroke risk in AF, reinforcing the contribution of prothrombotic and endothelial injury pathways to thromboembolic complications in this population [120].

#### Limitations of Blood Biomarkers

Several biomarkers lack disease specificity and may simply reflect systemic illness or advanced cardiovascular pathology [121,122]. Growth differentiation factor 15 (GDF-15), for example, is a stress-inducible cytokine implicated in a range of cardiovascular conditions and has been proposed as a prognostic marker for bleeding risk in patients with AF [123]. However, elevated GDF-15 levels are also associated with increased mortality and major adverse cardiovascular events (MACEs) in individuals without AF, highlighting its limited specificity [124].

Practical barriers such as assay variability, diurnal fluctuations in biomarker concentrations, and cost-related constraints further challenge the routine clinical implementation of both blood- and urine-based biomarkers [125]. As such, a careful balance must be struck between the added prognostic insight offered by these markers and the need for operational simplicity in high-volume clinical environments. To date, no biomarker has been prospectively validated to guide clinical decision-making in the management of AtCM.

### 3.2. Mult-Omics, AF, and AtCM

Primary AtCM may originate from pathogenic variants in genes with either atrial-specific expression or broader systemic functions that manifest within the atrial myocardium [126,127]. Among atrial-selective genetic determinants, MYL4, encoding the essential myosin light chain specific to atrial tissue, has been linked to profound electromechanical abnormalities and atrial contractile dysfunction [128,129]. Similarly, mutations in NPPA, which encodes atrial natriuretic peptide (ANP), have been associated with marked biatrial dilation, heightened thromboembolic risk, and atrial standstill syndromes [126,130]. In addition, several gene variants traditionally associated with ventricular or multisystem disorders have been implicated in atrial dysfunction. These include HCN4, responsible for the cardiac pacemaker channel [131]; SCN5A, which encodes the primary voltage-gated sodium channel in cardiomyocytes [132]; SCN1B, encoding the β1/β1B subunits of the sodium channel complex [133]; and LMNA, which encodes the nuclear envelope protein Lamin A/C [134].

Current understanding of the genomic, transcriptomic, and epigenomic landscape of AtCM is largely extrapolated from investigations focused on AF, a condition that itself contributes to atrial structural and functional impairment. Large-scale genome-wide association studies (GWASs) have identified numerous chromosomal loci containing common genetic variants associated with atrial electrophysiological traits, including heart rate, P-wave duration, and the PR interval [135]. Additional variants have been linked to cardiac anatomical and functional characteristics, such as chamber size, myocardial structure, and contractility [136]. Collectively, GWASs have uncovered more than 100 genetic loci associated with increased susceptibility to AF [137,138]. While it remains unclear whether these variants directly drive the development of primary AtCM, their established association with AF suggests a potential role in promoting secondary atrial remodeling.

Transcriptomic analyses of human atrial tissue—employing both microarray and RNA sequencing technologies—have uncovered a range of genes implicated in atrial remodeling processes [139]. Among these are NPPA and NPPB, which encode natriuretic peptides and are well-established markers of myocardial stress [140], as well as GPR22 and RGS6, involved in G-protein–mediated signaling pathways. Other notable findings include NTM, encoding neurotrimin, a novel biomarker associated with heart failure [141], and COLQ, expressed in both atria, which encodes a specialized collagen responsible for anchoring acetylcholinesterase to the basal lamina, suggesting neuromodulatory involvement in atrial pathology [142].

A recent mRNA sequencing study of atrial tissue obtained during cardiac surgery identified 35 genes significantly associated with AF, independent of coexisting heart failure [140]. These genes were functionally enriched in pathways regulating cardiomyocyte architecture, electrical conduction, fibrosis, inflammation, and endothelial dysfunction, highlighting the multifaceted nature of the arrhythmogenic substrate [143]. Furthermore, in a large-animal model of AF progression, substantial transcriptomic and proteomic alterations were observed early in the transition from paroxysmal to persistent AF, indicating that molecular remodeling precedes sustained arrhythmogenesis [144].

It is now well established that AF arises from the complex interplay between genetic predisposition and environmental influences [145]. Notably, the majority of AF-associated loci identified through GWAS are located within non-coding regions of the genome [146], where they exert regulatory effects on gene expression by modulating transcription factor binding and altering chromatin accessibility through epigenetic mechanisms. Epigenetics, defined as heritable changes in gene expression that do not involve alterations to the DNA sequence itself, has emerged as a key regulatory layer in AF pathophysiology [146].

Among the epigenetic modifications implicated in AF, DNA methylation of CpG islands—mediated by DNA methyltransferases (DNMTs)—has been observed in both paroxysmal and persistent forms of the arrhythmia [147,148]. A recent study employed four DNA methylation-based biological age estimators to provide an epigenetic rationale for the established relationship between chronological aging and AF susceptibility, further bridging aging-related processes with atrial remodeling [149,150].

Beyond DNA methylation, histone modifications have also been implicated in AF pathogenesis [151,152]. Enzymes such as histone deacetylases (HDACs) and histone acetyltransferases (HATs) have been shown to modulate transcriptional activity in atrial tissue, with dysregulation contributing to electrical and structural remodeling [151]. In patients with AF, increased expression of EZH2, which encodes a histone methyltransferase responsible for catalyzing the repressive H3K27me3 epigenetic mark, has been documented in both atrial cardiomyocytes and fibroblasts [152].

### 3.3. ECG Abnormalities in AtCM

In AtCM, the underlying structural, architectural, contractile, and electrophysiological remodeling of the atria is reflected in distinct abnormalities on surface electrocardiography, serving as a non-invasive manifestation of atrial pathology [153,154].

Atrial remodeling is frequently accompanied by electrocardiographic manifestations, particularly alterations in P-wave duration and morphology, which reflect underlying conduction disturbances. A P-wave duration ≥ 120 ms is indicative of partial interatrial block (IAB), while a biphasic P-wave morphology in the inferior leads (II, III, and aVF) signifies advanced IAB, typically caused by complete conduction block at Bachmann’s bundle [153,154,155]. IAB, particularly in its advanced form, is closely associated with LA enlargement [156] and has been linked to the presence of atrial fibrosis, a substrate conducive to reentrant activity, ectopic triggering, and ultimately the initiation and perpetuation of AF [157,158].

Patients exhibiting prolonged P-wave duration or advanced IAB are at heightened risk for AF recurrence following catheter ablation [159,160]. Interestingly, abnormally short P-wave durations have also been identified as predictors of both incident [161] and recurrent AF [162], suggesting that deviations in atrial conduction time at either extreme may reflect distinct forms of electrical remodeling.

High-resolution digital analysis of P-wave duration—typically using amplification settings of 150–200 mm/sec and 80–100 mm/mV—has been proposed as a valuable non-invasive marker for stratifying the severity of AtCM. Specific thresholds of amplified P-wave duration (APWD) correspond to discrete AtCM stages: 140–150 ms for early-stage disease, 150–180 ms for moderate AtCM, and >180 ms or the presence of biphasic P-waves in ≥2 inferior leads for advanced stages [163,164,165]. These electrocardiographic phenotypes have been associated with reduced LAA flow velocities, increased thrombogenic potential, and elevated risk for major adverse cardiovascular and cerebrovascular events, underscoring their clinical relevance [163,164,165].

In addition to P-wave duration, P-wave terminal force in lead V1 (PTFV1) has emerged as a widely utilized electrocardiographic marker of AtCM. Calculated as the product of the terminal duration (PTDV1) and the absolute amplitude (PTAV1) of the terminal negative P-wave deflection in V1, a PTFV1 exceeding 4 mV·ms is considered pathological [166]. Abnormal PTFV1 has been linked to impaired LAA contractile function, as evidenced by reduced ejection velocities on TEE [167]. While initially interpreted as a marker of LA enlargement and posterior anatomical displacement, more recent evidence suggests that abnormal PTFV1 reflects altered conduction patterns, particularly delayed and posteriorly directed terminal activation of the LA [168,169].

The presence of an abnormal PTFV1 has also been associated with reduced LA strain on speckle-tracking echocardiography, indicating a strong relationship with functional atrial remodeling [169]. A recent meta-analysis confirmed that pathological PTFV1 is predictive of AF occurrence in both general and cardiovascular populations and serves as a prognostic marker for arrhythmia recurrence following CA [170,171]. Furthermore, advanced ECG analysis using amplified digital 12-lead recordings has shown that prolonged P-wave duration is significantly associated with low-voltage substrate in the LA, providing a non-invasive surrogate for fibrotic remodeling [172]. This approach has demonstrated utility in identifying patients with persistent AF at high risk for recurrence after CA and has also been linked to the development of new-onset AF in individuals with HFpEF [173].

The frontal P-wave axis, normally ranging between 0° and +75°, serves as an indicator of the overall direction of atrial depolarization [153]. Deviations from this range have been associated with increased susceptibility to AF. A recent meta-analysis demonstrated that an abnormal P-wave axis is predictive of future AF detection, reinforcing its utility as a non-invasive marker of atrial electrical remodeling [166].

Similarly, reduced P-wave voltage—specifically a P-wave amplitude ≤ 0.1 mV in lead I—is considered pathological [153]. Incorporation of low P-wave voltage into predictive scoring systems has shown utility in identifying individuals at risk for developing AF [174]. Moreover, attenuated P-wave amplitude in lead I has been independently associated with arrhythmia recurrence following radiofrequency ablation, suggesting that diminished atrial electrical signal strength may reflect a pro-arrhythmic substrate [175].

The P-wave area, calculated in lead II as ½ × P-wave duration × P-wave voltage [153], provides an integrative measure of atrial depolarization magnitude. A value ≥ 4 ms·mV is considered abnormal and has been associated with LA enlargement, reflecting structural atrial remodeling [176]. In specific clinical contexts, such as mitral stenosis, the P-wave area measured in lead V3 has demonstrated predictive value for new-onset AF, suggesting its applicability in disease-specific risk stratification [177]. P-wave dispersion, defined as the difference between the maximum and minimum P-wave durations across the 12-lead ECG, serves as a surrogate for intra-atrial conduction heterogeneity [153]. Increased P-wave dispersion has been linked to a higher risk of both incident AF and arrhythmia recurrence following cardioversion [178]. Moreover, in patients with cryptogenic stroke monitored by implantable loop recorders, a P-wave dispersion exceeding 40 ms has been identified as a significant predictor of subsequent AF detection, underscoring its relevance in subclinical AF surveillance [179].

Fibrillatory wave (f-wave) characteristics on surface ECG offer valuable insights into the underlying atrial substrate in AF. Diminished f-wave amplitude has been consistently associated with increased LA size and reduced LAA flow velocity, suggesting a correlation between attenuated atrial electrical activity and structural remodeling [180,181]. Distinct f-wave patterns also demonstrate diagnostic and prognostic relevance. Specifically, low-amplitude f-waves in lead II combined with elevated dominant frequencies in lead V1 have been shown to differentiate long-standing persistent AF from paroxysmal or short-duration persistent forms, indicating a more advanced electrophysiological phenotype [182]. In community-based cohorts, the presence of fine rather than coarse f-waves has been observed more frequently in individuals with persistent AF or greater LA enlargement and independently correlates with elevated risk of incident HF [183]. Moreover, lower pre-ablation f-wave amplitudes have been identified as predictors of post-procedural AF recurrence [184,185], reinforcing the utility of f-wave analysis as a non-invasive marker of advanced atrial disease and procedural outcome.

Advancements in ECG signal processing, particularly through signal-averaged P-wave analysis, have significantly enhanced the resolution and prognostic utility of surface electrocardiography in the context of atrial cardiomyopathy. Unlike standard 10 s ECGs, this approach involves the continuous recording and averaging of P-wave morphology over extended periods, thereby yielding more precise and reproducible data on atrial activation patterns. Prolonged signal-averaged P-wave durations have been consistently observed in individuals with paroxysmal AF compared to controls without a history of arrhythmia [186], and are also predictive of arrhythmia recurrence following catheter ablation [187]. Furthermore, parameters derived from prolonged ECG monitoring have demonstrated superior discriminatory capacity in differentiating paroxysmal from persistent AF relative to conventional short-duration recordings [188], underscoring their potential value in refining atrial substrate characterization and risk stratification strategies.

Finally, premature atrial contractions (PACs) have emerged as clinically relevant electrophysiological markers associated with atrial cardiomyopathy, heightened susceptibility to AF, and elevated risk of thromboembolic events [189,190]. Multiple studies have demonstrated that an increased PAC burden reflects underlying atrial electrical instability and may serve as an early indicator of pathological atrial remodeling [189,190].

#### Artificial Intelligence-Based ECG Analysis in AtCM

Artificial intelligence (AI)-driven analysis of P-wave morphology, including wavelet-based techniques, represents a promising frontier in the electrocardiographic assessment of atrial cardiomyopathy [153,191]. Emerging deep learning models have demonstrated the capacity to identify individuals at elevated risk for AF with high precision, even when ECGs are recorded in sinus rhythm [192], suggesting that traditional P-wave metrics may insufficiently capture the full spectrum of atrial electrical abnormalities.

In a pragmatic trial evaluating the utility of AI-guided ECG screening, individuals classified as high-risk by the AI algorithm exhibited a fivefold increase in AF detection rates compared to those in the low-risk group [193], underscoring the potential of AI to enhance the sensitivity and efficiency of population-based AF screening strategies. Beyond risk prediction, AI methodologies have also been applied successfully to quantify the atrial substrate and to identify electrophysiological targets for catheter ablation [194,195], highlighting their expanding role in both diagnostic and therapeutic dimensions of atrial disease management.

## 4. Conclusions and Future Directions

The concept of AtCM has redefined AF as a consequence of progressive structural, electrical, contractile, and molecular remodeling of the atria. This paradigm shift necessitates diagnostic approaches that extend beyond rhythm classification to capture the underlying substrate driving arrhythmogenesis. As reviewed herein, advances in multimodality imaging, electroanatomic mapping, biomarker profiling, ECG analysis, and omics technologies have enabled a more comprehensive assessment of AtCM, offering novel opportunities for early detection, refined risk stratification, and individualized therapy.

Despite these advances, substantial gaps remain. Imaging modalities such as CMR and CCT have improved visualization of atrial fibrosis and morphology, yet standardization of acquisition protocols, quantification thresholds, and clinical integration remain limited. ECG-derived metrics—including P-wave duration, morphology, dispersion, and f-wave characteristics—hold promise as accessible markers of electrical remodeling but require further validation in prospective studies. Similarly, biomarker-based definitions of AtCM remain challenged by limited specificity and variability across populations. The integration of omics approaches, while conceptually powerful, has yet to yield clinically actionable phenotypes distinct from AF-driven remodeling.

The future of AtCM characterization lies in harmonizing these diverse diagnostic platforms to enable early, precise, and clinically meaningful identification of atrial disease. Key priorities include the following: (i) establishing standardized, reproducible criteria for AtCM across imaging, ECG, and biomarker domains; (ii) elucidating substrate-specific predictors of stroke, heart failure, and arrhythmia recurrence; (iii) validating AI-enhanced tools for non-invasive mapping and risk prediction; and (iv) designing interventional trials that incorporate AtCM phenotypes to guide therapy selection.

Ultimately, embedding substrate characterization into clinical decision-making may transform the contemporary management of AF—from reactive treatment of arrhythmia to proactive modification of its underlying disease substrate.

## Figures and Tables

**Table 1:** Diagnostic utility of multimodality imaging in atrial structural and functional assessment.

Atrial Parameters	TTE	TEE	Cardiac MRI	Cardiac CT	Electroanatomic Mapping ^a^
Atrial size	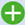 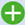 (more accurate in 3D TTE)	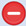	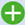 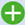	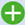 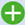 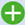	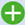
Atrial function	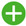 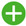 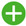 (more accurate in 3D TTE)	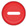	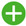 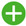 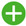	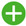	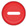
Atrial appendage size	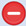	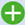 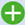 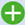	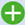	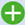 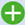 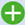	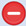
Thrombus	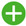	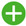 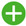 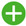	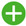 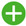	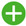 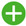 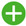 (delayed acquisition) ^b^	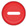
Epicardial adipose tissue	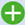	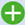	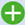	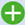 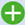 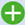	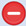
Atrial fibrosis	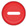	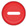	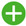 (LGE) ^b^	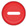	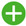
Atrial velocity	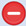	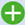 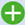 (exclusively in atrial appendage)	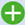 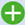 (4D flow imaging)	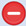	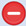
Atrial activation/conduction time	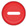	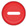	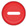	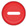	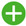 (P-wave duration)

Abbreviations: 3D, three-dimensional; CT, computed tomography; LGE, late gadolinium enhancement; MRI, magnetic resonance imaging; TEE, transoesophageal echocardiography; TTE, transthoracic echocardiography. ^a^ Electroanatomic mapping comprises a comprehensive array of electrophysiological techniques, including voltage mapping, activation sequence reconstruction, and the analysis of conduction velocity and fractionated electrograms, collectively aimed at delineating atrial substrate complexity. ^b^ Administration of contrast agents is required to enhance image quality and enable accurate tissue characterization within the respective imaging protocols.

**Table 2:** Multimodal characterization of cardiac chamber structure and function in atrial fibrillation: prognostic utility of left atrial strain in stroke risk stratification.

Author, Year	Population	Methods of Evaluating Parameters	Results
Leung et al., 2017 [44]	1361 patients with first-diagnosed AF (mean age 65 ± 12 years; 74% male) followed over 7.9 years for stroke/TIA occurrence	TTE with 2D speckle tracking for LA reservoir, conduit, and booster pump strain; PA-TDI for total atrial conduction time; LV GLS and standard parameters	Reduced LA reservoir and conduit strain and prolonged PA-TDI were independently associated with stroke risk beyond CHA2DS2-VASc score, age, and anticoagulant use. LA volume and LVEF were not predictive.
Obokata et al., 2014 [28]	286 patients with nonvalvular AF: 82 with acute embolism (stroke or systemic embolism); 204 controls; prospectively followed	Speckle-tracking TTE to assess global peak LA longitudinal strain (LAS) during AF rhythm; LAVI, LA emptying fraction, and Doppler echocardiography; CHA2DS2-VASc score; outcome follow-up for mortality	Global LAS was significantly lower in patients with acute embolism. LAS < 15.4% identified embolism with AUC 0.83 and predicted mortality post-embolism. LAS was independently associated with embolism risk and added incremental value over CHA2DS2-VASc. LA volumes were not predictive.
Azemi et al., 2012 [29]	57 patients with AF, stroke/TIA, and CHADS2 ≤ 1; compared with matched controls without stroke or TIA	TTE with velocity vector imaging to assess peak positive and negative LA strain and strain rate; LAVI, LA dimension, and LVEF; binary logistic regression for outcome prediction	Both peak positive and negative LA strain were significantly reduced in patients with stroke/TIA. These strain indices were independently associated with stroke/TIA risk, outperforming conventional metrics such as LAVI and LVEF.
Shih et al., 2011 [24]	66 patients with permanent AF, 20 with previous ischemic stroke, and 46 without stroke	TTE with 2D speckle tracking to assess peak positive LA strain (LASp), reservoir strain rate (LASRr), and conduit strain rate (LASRc); standard echocardiographic measures (LAVI, LATEF, and E/E’); multivariate logistic regression	LASp and LASRr were significantly lower in stroke group and independently associated with stroke after adjusting for age, LAVI, and LVEF. LASp < 13.5% had 80% sensitivity and 63% specificity for stroke. LAVI and LATEF were not predictive.

Abbreviations: AF, atrial fibrillation; AUC, area under the curve; CI, confidence interval; E/E′, ratio of early mitral inflow velocity to early diastolic mitral annular velocity; GLS, global longitudinal strain; LA, left atrium; LAVI, left atrial volume index; LAS, left atrial strain; LASp, peak positive left atrial strain; LASRc, left atrial strain rate during the conduit phase; LASRr, left atrial strain rate during the reservoir phase; LATEF, left atrial total emptying fraction; LVEF, left ventricular ejection fraction; OR, odds ratio; PA-TDI, total atrial conduction time by tissue Doppler imaging; TIA, transient ischemic attack; TTE, transthoracic echocardiography.

## Data Availability

All data generated in this research are included within this article.
